# A risk stratification and prognostic prediction model for lung adenocarcinoma based on aging-related lncRNA

**DOI:** 10.1038/s41598-022-26897-2

**Published:** 2023-01-10

**Authors:** HuiWei Chen, Lihua Peng, Dujuan Zhou, NianXi Tan, GenYi Qu

**Affiliations:** 1 Department of Emergency, Zhuzhou Central Hospital, Zhuzhou, 412007 Hunan China; 2 Department of Otolaryngology Head and Neck Surgery, Zhuzhou Central Hospital, Zhuzhou, 412007 Hunan China; 3 Department of Teaching, Zhuzhou Central Hospital, Zhuzhou, 412007 Hunan China; 4 Department of Cardiothoracic Vascular Surgery, Zhuzhou Central Hospital, Zhuzhou, 412007 Hunan China; 5 Department of Urology, Zhuzhou Central Hospital, Zhuzhou, 412007 China

**Keywords:** Cancer, Computational biology and bioinformatics

## Abstract

To create a risk model of aging-related long non-coding RNAs (arlncRNAs) and determine whether they might be useful as markers for risk stratification, prognosis prediction, and targeted therapy guidance for patients with lung adenocarcinoma (LUAD). Data on aging genes and lncRNAs from LUAD patients were obtained from Human Aging Genomic Resources 3 and The Cancer Genome Atlas, and differential co-expression analysis of established differentially expressed arlncRNAs (DEarlncRNAs) was performed. They were then paired with a matrix of 0 or 1 by cyclic single pairing. The risk coefficient for each sample of LUAD individuals was obtained, and a risk model was constructed by performing univariate regression, least absolute shrinkage and selection operator regression analysis, and univariate and multivariate Cox regression analysis. Areas under the curve were calculated for the 1-, 3-, and 5-year receiver operating characteristic curves to determine Akaike information criterion-based cutoffs to identify high- and low-risk groups. The survival rate, correlation of clinical characteristics, malignant-infiltrating immune-cell expression, ICI-related gene expression, and chemotherapeutic drug sensitivity were contrasted with the high- and low-risk groups. We found that 99 DEarlncRNAs were upregulated and 12 were downregulated. Twenty pairs of DEarlncRNA pairs were used to create a prognostic model. The 1-, 3-, and 5-year survival curve areas of LUAD individuals were 0.805, 0.793, and 0.855, respectively. The cutoff value to classify patients into two groups was 0.992. The mortality rate was higher in the high-risk group. We affirmed that the LUAD outcome-related independent predictor was the risk score (p < 0.001). Validation of tumor-infiltrating immune cells and ICI-related gene expression differed substantially between the groups. The high-risk group was highly sensitive to docetaxel, erlotinib, gefitinib, and paclitaxel. Risk models constructed from arlncRNAs can be used for risk stratification in patients with LUAD and serve as prognostic markers to identify patients who might benefit from targeted and chemotherapeutic agents.

## Introduction

Lung cancer is a lethal malignancy associated with substantial morbidity and mortality. Lung adenocarcinoma (LUAD) is the predominant type of non-small cell lung cancer^1^. Although molecularly targeted therapy combined with chemotherapy has achieved remarkable results, the 5-year overall survival rate does not exceed 15%^2^. It is critical to develop a new model for risk classification and prognosis prediction in LUAD patients and to facilitate individualized treatment.

Aging is a natural process in which biological functions decline over time. Advanced age is a risk factor for cancer and is associated with a high risk of cardiovascular, metabolic, neoplastic, and neurodegenerative diseases^3–5^. Aging is related to gene instability, cell decline, and secretion of harmful substances from aging genes that can cause irreversible damage^6,7^. The fundamental pathogenetic step is gene mutation^8^ which causes cells to divide indefinitely, followed by freedom from the monitoring of immune cells.

Long non-coding RNAs (lncRNAs) represent about 80% of the human transcriptome. They modulate genes by interacting with RNAs and proteins to enhance or repress expression^9^. They can also lead to malignant phenotypes of cancer through the immune microenvironment^10^. LncRNAs modulate various biological processes essential in normal human development and tumorigenesis, including autophagy, apoptosis, and necroptosis. LncRNAs participate in immune evasion and aggravation of inflammation in malignant tumors^11^. Xu et al. developed a model based on an aging-related gene signature and other clinical parameters (which might anticipate outcomes of individuals with LUAD) and utilized it to manage LUAD patients^12^. Zhang et al. showed that signatures of aging-related lncRNAs can determine glioma patients’ prognoses and suggest individualized treatment strategies^13^.

Aging-associated lncRNAs may have potential value in risk stratification and prognosis in patients with LUAD and serve as potential therapeutic targets for treatment. This investigation aimed to establish and validate this aging-related lncRNA prognostic model, which could better serve for clinical diagnosis and patient treatment.

## Materials and methods

### Data acquisition

Transcriptome analysis (RNAseq) data, and related clinical data of LUAD, were retrieved from The Cancer Genome Atlas (TCGA) website (https://tcga-data.nci.nih.gov/tcga/). We downloaded gene transfer format files from Ensembl (http://asia.ensembl.org) to differentiate between mRNAs and lncRNAs; these files were converted to official gene symbols, and the data were log2 processed. LncRNAs were identified using the Ensembl Human Genome Browser GRCh38^14^.

### Identification of aging-related lncRNAs

A profile of 307 aging-related genes was obtained from the Human Aging Genomic Resources 3 (Supplementary Table 1). Using Pearson's correlation analysis and co-expression strategy, lncRNAs with co-expression correlation coefficients > 0.7 and p-values < 0.001 were defined as aging-related long non-coding RNAs (arlncRNAs). ArlncRNAs with log fold-change > 1.0 and false discovery rate < 0.05 were identified as differentially expressed arlncRNAs (DEarlncRNAs).

### Construction of DEarlncRNA pairs

Multiple rounds of cyclic pairing were performed on DEarlncRNA; α = 1 indicates that the level of expression of lncRNA A is significantly elevated compared with lncRNA B samples, while α = 0 indicates that the expression level of lncRNA A is lower than lncRNA B samples. Then a 0-or-1 matrix was created for subsequent filtering. Correct prediction of survival outcomes requires a specific level of pairs. We defined effective pairing as the sum of lncRNA pairs with an expression level of 0 or 1, accounting for > 20% and < 80% of the total pairs.

### Establishment of the risk model

We merged the arlncRNA pairs with the downloaded survival data from LUAD patients, removed duplicate data and data with insufficient follow-up, and obtained arlncRNA pairs that were closely related to prognosis utilizing univariate regression analysis (screening criterion was p < 0.05). To prevent overfitting, we performed least absolute shrinkage and selection operator (LASSO) regression analysis on the initially selected arlncRNA pairs, repeated the random method 1000 times, and performed the second cross-validation on the arlncRNA pairs whose matching frequency exceeded 100 times (p < 0.05). Finally, we chose the best pairing combination to construct the risk model of arlncRNA and obtained the formula for the model. The risk score equals the expression level of every arlncRNA pair multiplied by the risk factor. The risk value for each LUAD sample was equivalent to the aggregate expression levels of every arlncRNA pair multiplied by the corresponding risk factor^9^.

$$\mathrm{Risk \, Score }={\sum }_{i=1}^{n}\mathrm{Risk \, coefficient \, }i\times \mathrm{arlncRNA \, Expression} i$$

Based on the risk score, we computed the area under the curve (AUC) for every model and plotted the ROC curves using the “survROC” package (version 4.1.3). The ROC curves permitted the selection of the optimal cutoff to categorize the patients into high- and low-risk groups.

### Clinical correlation analysis of the risk model

Kaplan–Meier curves were constructed to determine whether there was a difference in survival between the high- and low-risk groups. p < 0.05 denoted a statistically substantial difference, and there was variation in survival between the groups. The link between risk scores and clinical indicators (i.e., sex, survival status, age, stage, and T, N, and M stage) was analyzed utilizing the chi-square test. The Wilcoxon rank-sum test evaluated associations between risk scores and clinical index subgroups. Using univariate and multivariate Cox regression analysis, we determined whether we could utilize the risk model as an independent prognostic factor and as a model independent of these other clinical traits.

### Correlation analysis of tumor-infiltrating immune cells

Techniques for analyzing the link between risk scores in conjunction with immune-cell infiltration are based on XCELL (http://xCell.ucsf.edu/)^15^, QUANTISEQ (http://icbi.at/quantiseq)^16^, TIMER (Version 2.0; http://timer.cistrome.org/)^17^, CIBERSORT (http://cibersort.stanford.edu/)^18^, EPIC (http://epic.gfellerlab.org)^19^, MCPCOUNTER and CIBERSORT-ABS. Wilcoxon signed-rank test, limma, scales, ggplot2, and ggtext R packages were performed in the course of implementation.

### Immunosuppressive molecules expression analysis related to ICIs

The “limma (version 3.0)” and “ggpubr (version 3.2.1)” packages in R were utilized to determine whether there were significant differences in gene expression that are ICI-related between the two groups.

### Correlation analysis of chemotherapeutic agents

We selected conventional chemotherapy drugs for lung adenocarcinoma (erlotinib, cisplatin, paclitaxel, docetaxel, gefitinib) to determine whether there was a difference in the response of the groups of LUAD patients to chemotherapeutic drugs (alone or in combination). We used the drug's 50% inhibition rate (IC_50_) to measure drug sensitivity. Lower IC_50_ indicates greater sensitivity. The R package pRRophetic was utilized to evaluate their therapy response.

## Results

### Differentially expressed aging-related lncRNAs in LUAD patients

**Figure 1:**
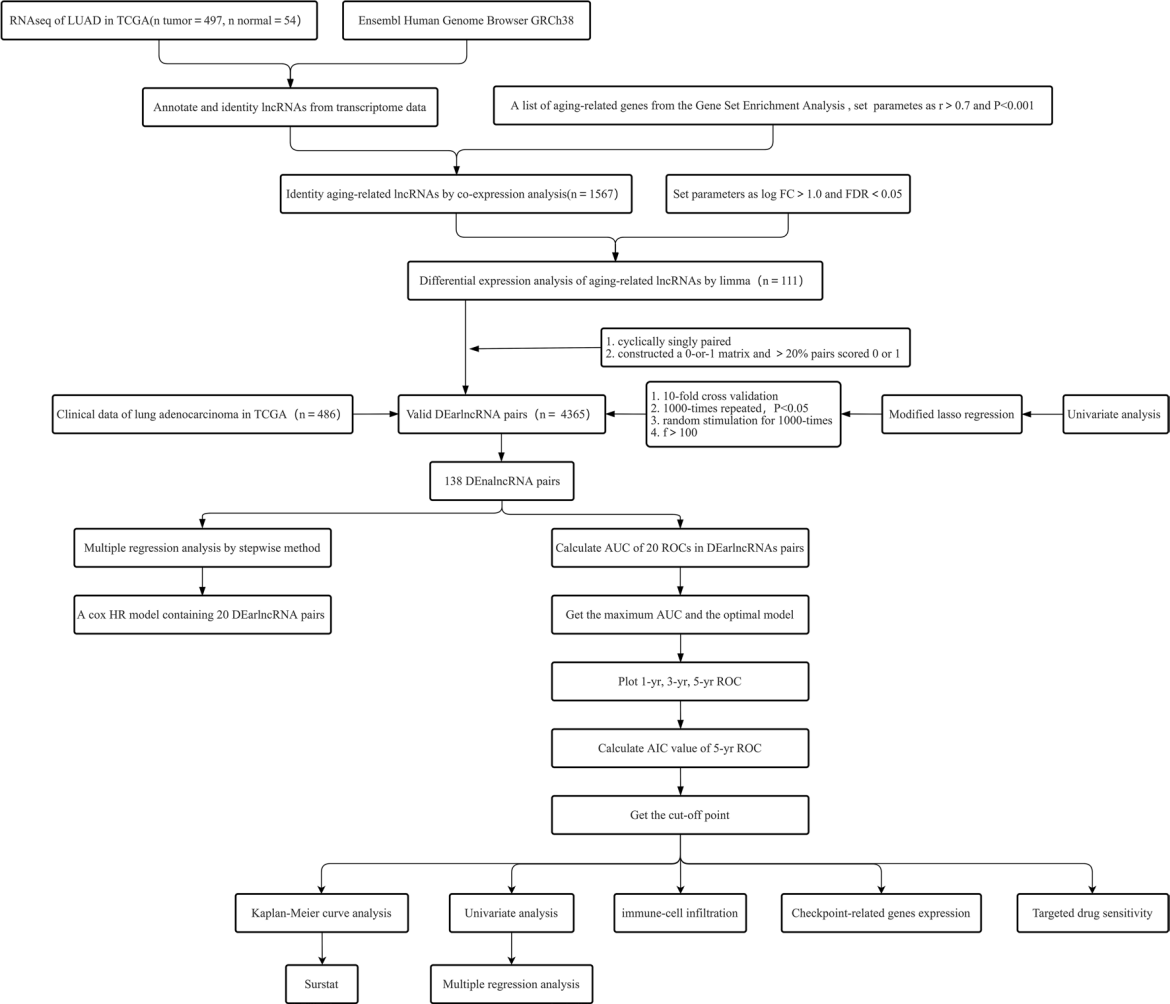
The study’s flow chart.

**Figure 2:**
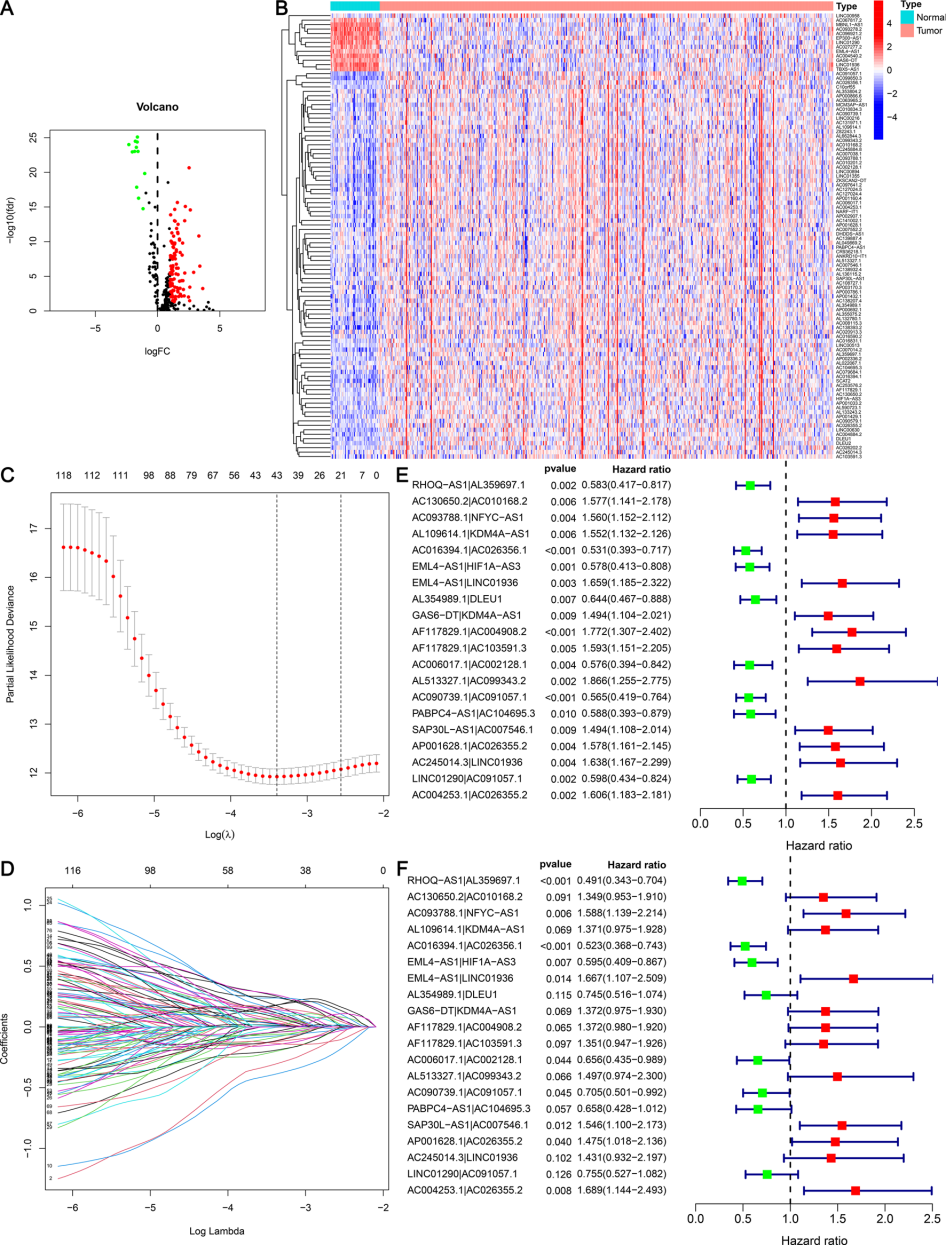
Differential expression analysis of aging-associated lncRNAs in LUAD and generated heatmaps and volcano plots. (**A**) Volcano plot. Red dots: Elevated expression levels of lncRNAs; green dots: downregulated lncRNAs; black dots: lncRNAs with no substantial difference. (**B**) Heatmap. Azure: samples from the normal group; orange: samples from the tumor group; red: high expression of aging-related lncRNAs; blue: low expression of aging-related lncRNAs. The “pheatmap (version 4.13)” packages in R was used to draw heatmap. We downloaded the R software (version 4.13) from the official website (https://mirrors.tuna.tsinghua.edu.cn/CRAN/). (**C**) The LASSO coefficient profile of 20 aging-related lncRNAs. (**D**) The cross-validation for variable selection in the LASSO model. (**E**) Forest plot of Cox univariate regression analysis. (**F**) Forest plot of Cox multivariate regression analysis. Red: Risk factors; green: protective factors.

The research process is depicted in a flowchart (Fig. 1). We retrieved LUAD transcriptome data from TCGA and included 497 LUAD samples and 54 paracancerous tissue samples. We obtained a profile of 307 aging-related genes from the Human Aging Genomic Resources 3 (Supplementary Table 1). We identified 1567 arlncRNAs (Supplementary Table 2) by co-expressing the acquired lncRNAs and aging-related genes. Through differential analysis, we obtained 111 DEarlncRNAs, of which 99 were upregulated, and 12 were downregulated. We then drew heat and volcano maps (Fig. 2A,B and Supplementary Table 3).

### Differentially expressed aging-related lncRNA pairs and risk models

After several rounds of the cyclic pairing of 111 DEarlncRNAs, 4365 pairs of differentially expressed aging-related lncRNA were obtained (Supplementary Table 4). Univariate Cox regression analysis revealed that 138 DEarlncRNA pairs were associated with the prognosis of LUAD patients (Supplementary Table 5). LASSO regression analysis (Fig. 2C,D) identified 20 DEarlncRNAs pairs to construct a risk model. Multivariate and univariate Cox regression analysis (Fig. 2E,F) revealed 20 pairs of prognostic aging-related lncRNAs multivariate Cox regression results (Table 1).

**Table 1:** 20 pairs of prognostic aging-related lncRNA pairs multivariate COX regression analysis results.

LncRNAs	Coefficient	HR	HR.95L	HR.95H	P-value
RHOQ-AS1|AL359697.1	− 0.7110	0.4912	0.3428	0.7038	0.0001
AC130650.2|AC010168.2	0.2996	1.3493	0.9530	1.9103	0.0913
AC093788.1|NFYC-AS1	0.4626	1.5881	1.1392	2.2139	0.0064
AL109614.1|KDM4A-AS1	0.3157	1.3712	0.9752	1.9278	0.0694
AC016394.1|AC026356.1	− 0.6480	0.5231	0.3682	0.7430	0.0003
EML4-AS1|HIF1A-AS3	− 0.5189	0.5951	0.4087	0.8666	0.0068
EML4-AS1|LINC01936	0.5108	1.6666	1.1070	2.5091	0.0144
AL354989.1|DLEU1	− 0.2950	0.7445	0.5161	1.0740	0.1146
GAS6-DT|KDM4A-AS1	0.3164	1.3721	0.9753	1.9304	0.0693
AF117829.1|AC004908.2	0.3163	1.3720	0.9803	1.9203	0.0652
AF117829.1|AC103591.3	0.3006	1.3507	0.9470	1.9264	0.0971
AC006017.1|AC002128.1	− 0.4220	0.6557	0.4345	0.9895	0.0444
AL513327.1|AC099343.2	0.4033	1.4968	0.9742	2.2997	0.0656
AC090739.1|AC091057.1	− 0.3498	0.7048	0.5006	0.9924	0.0451
PABPC4-AS1|AC104695.3	− 0.4186	0.6580	0.4277	1.0122	0.0568
SAP30L-AS1|AC007546.1	0.4359	1.5463	1.1002	2.1733	0.0121
AP001628.1|AC026355.2	0.3883	1.4745	1.0179	2.1359	0.0400
AC245014.3|LINC01936	0.3581	1.4307	0.9318	2.1965	0.1016
LINC01290|AC091057.1	− 0.2807	0.7553	0.5272	1.0821	0.1260

*HR* hazard ratio, *HR.95L* 95% CI lower limit, *HR.95H* 95% CI upper limit.

### Evaluation of risk models in the role of stratification and prognosis

**Figure 3:**
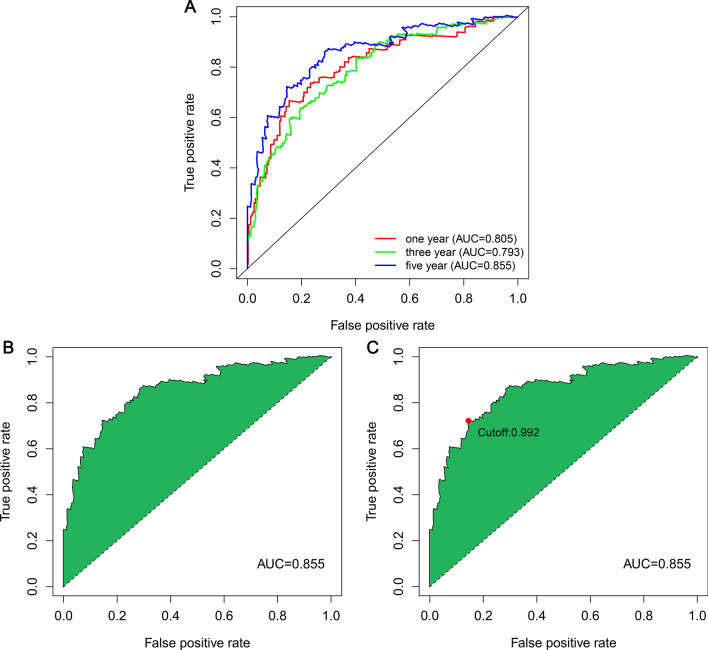
ROC curve based on the model value at risk. (**A**) The 1-, 3-, and 5-year ROC curves and AUCs of the optimal model. (**B**) The optimal ROC model corresponding to the maximum AUC. (**C**) The optimal cutoff value was 0.992, obtained by the Akaike information criterion and utilized to differentiate the high-risk and low-risk patients.

We plotted ROC curves for 1, 3, and 5 years, and we ascertained that the AUCs were all greater than 0.7, suggesting high prediction accuracy. The AUC for 5-years was 0.855, which was larger than the 1-year (AUC = 0.805) and 3-year (AUC = 0.793) (Fig. 3A). We used the Akaike information criterion to determine the optimal cutoff of 0.992 (Fig. 3B,C) and used this cutoff to differentiate the groups of LUAD patients. Ultimately, 234 and 230 patients were included in the low- and high-risk groups, respectively.

### Correlation analysis of clinical characteristics with the aid of risk models

**Figure 4:**
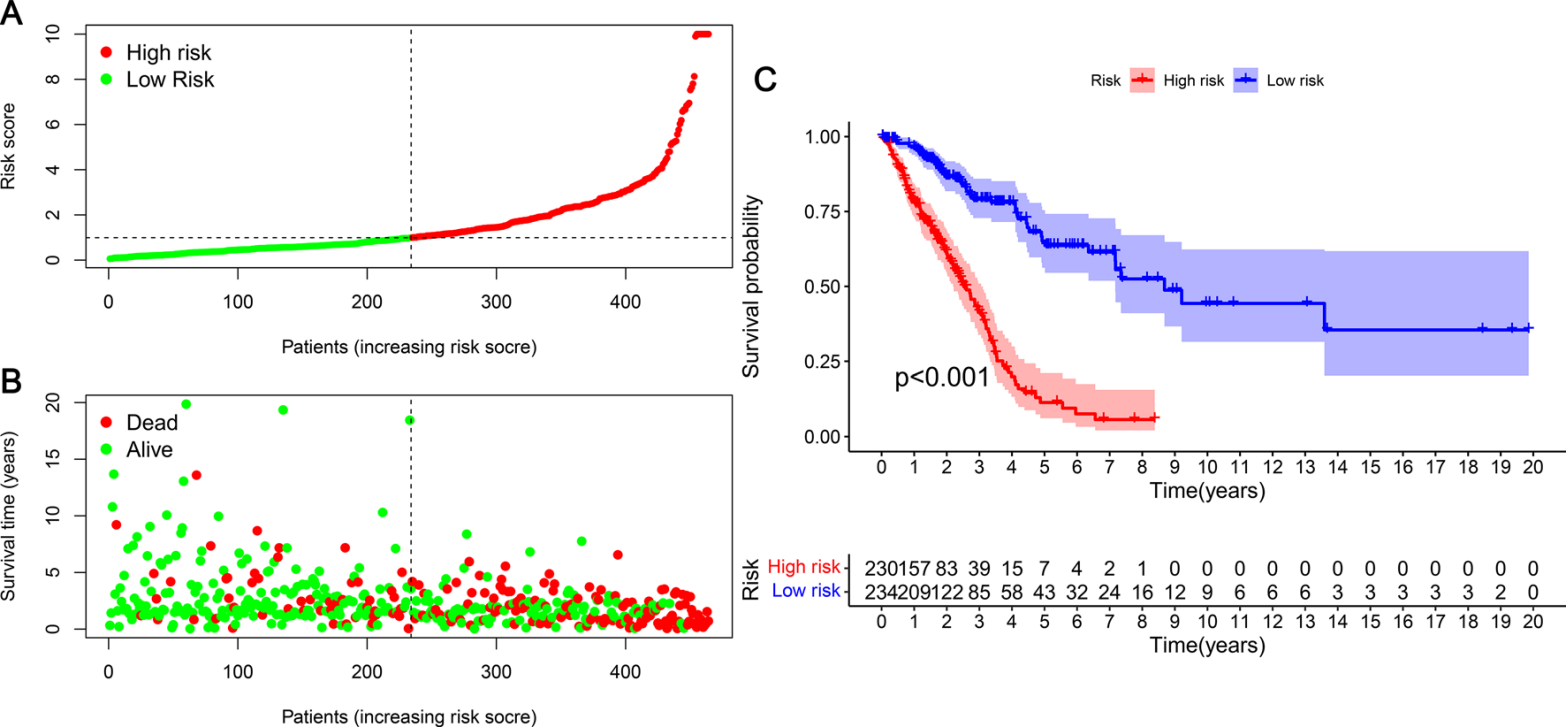
LUAD prognosis prediction risk model created by 20 aging-related lncRNAs. (**A**) Patients were categorized into low- and high-risk groups with the aid of risk scores. (**B**) Scatter plot of the relationship between patient risk scores and survival outcomes. (**C**) Kaplan–Meier curves were constructed to derive survival differences between high- and low-risk groups. Individuals in the low-risk group were found to have a longer survival time.

**Figure 5:**
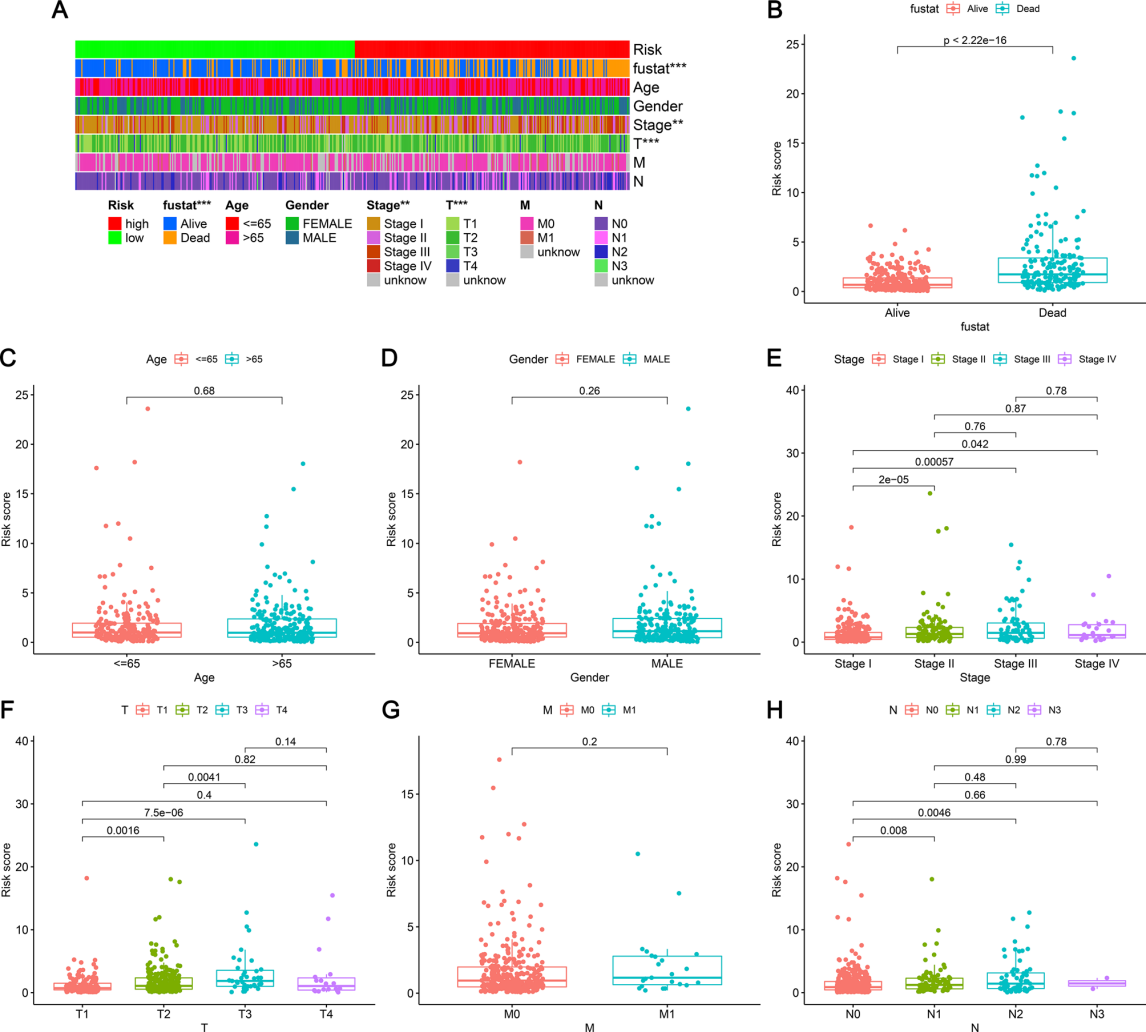
Risk factor model utilized for clinical association analysis in LUAD. (**A**) The clinical correlation strip chart. (**B**) survival status, (**C**) age, (**D**) gender, (**E**) Stage, (**F**) T stage, (**G**) M stage, (**H**) N stage.

The risk score and survival of every case are depicted in Fig. 4A and B. The survival in the low-risk group was significantly greater than that of the high-risk group. The Kaplan–Meier curve revealed that the survival of the low-risk group was significantly longer than the high-risk group (Fig. 4C) (p < 0.001). Utilizing chi-square and Wilcoxon signed-rank tests, we analyzed correlations between clinical features and LUAD risk using heat maps (Fig. 5A). There was a significant link between risk score and stage (p < 0.01), T stage (p < 0.001), and survival status (p < 0.001). Scatter plots of clinical features indicated that risk scores were significantly different for survival status (Fig. 5B), stage (Fig. 5E), and T stage (Fig. 5F). Age (Fig. 5C), gender (Fig. 5D), N stage (Fig. 5H), and M stage (Fig. 5G) were not significantly different. There were significant differences in risk scores between Stage I and II, Stage I and III, and Stage I and IV; however, there were no differences between Stage II, III, and IV. There were significant differences in risk scores between T1 and T2, T1 and T3, and T2 and T3.

**Figure 6:**
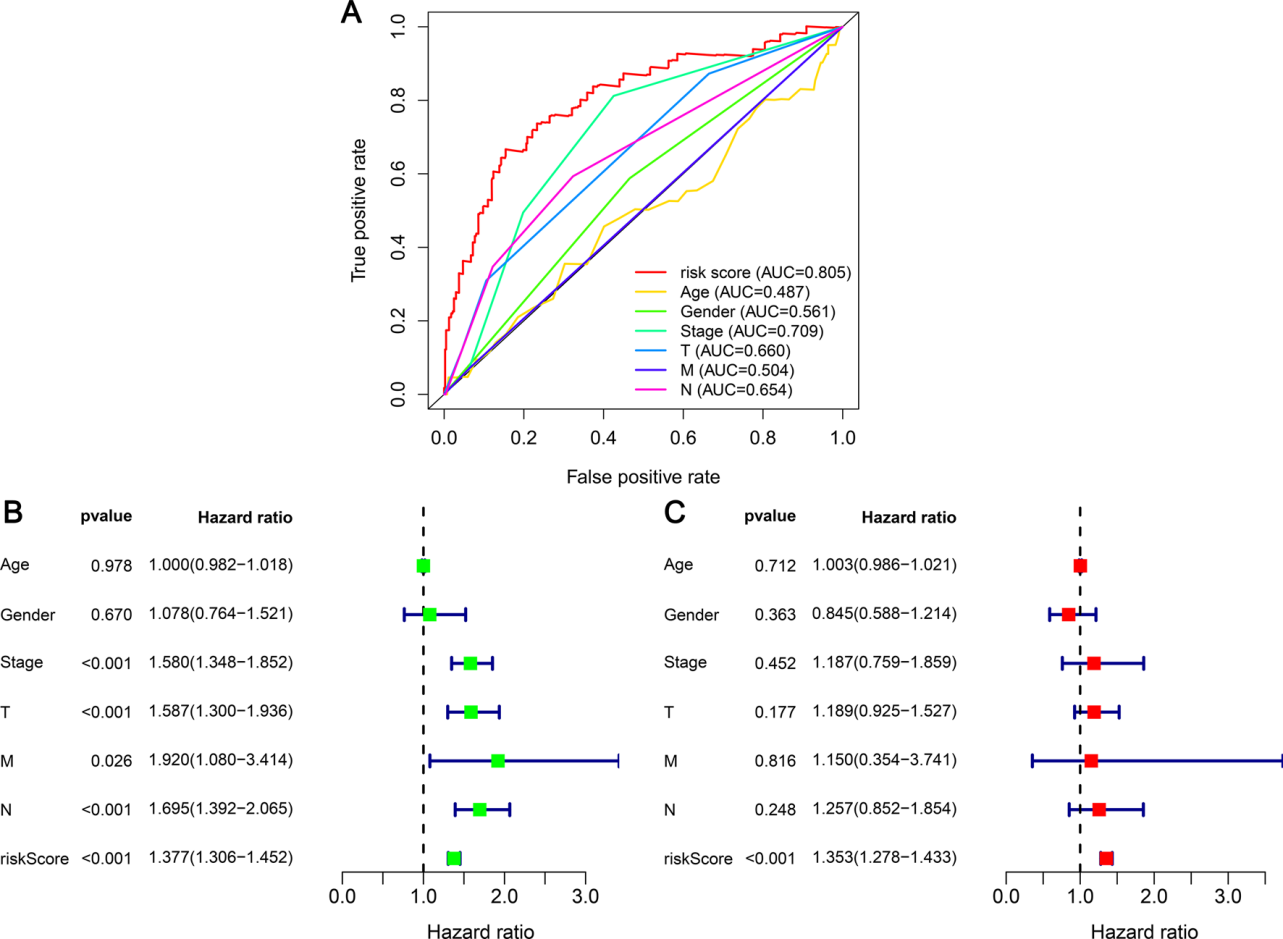
Cox regression analysis of clinically relevant characteristics and integrated ROC curves. (**A**) Riskscore (AUC = 0.805) and Stage (AUC = 0.709) had the greatest predictive performance. (**B**) Univariate Cox hazard ratio analysis demonstrated that Stage, T stage, N stage, and risk score were statistically different (p < 0.001). (**C**) Multivariate Cox analysis depicted the riskscore as the only independent predictor of prognosis (p < 0.001).

We compared ROC curves and risk factor scores for clinical characteristics over 1 year (Fig. 6A). Risk score (AUC = 0.805) and stage (AUC = 0.709) had the strongest predictive power. According to univariate Cox regression analysis, LUAD patients were classified by stage (p < 0.001, HR 1.580, 95% confidence interval [CI] [1.348–1.852]), T stage (p < 0.001, HR 1.587, 95% CI [1.300–1.936]), N stage (p < 0.001, HR 1.695, 95% CI [1.392–2.065]), and risk score (p < 0.001, HR 1.377, 95% CI [1.306–1.452]) (Fig. 6B)). However, multivariate Cox regression analysis found that risk score (p < 0.001, HR 1.353, 95% CI [1.278–1.433]) was the only independent predictor of prognosis (Fig. 6C).

### Correlation of immune-cell infiltration and risk model

**Figure 7:**
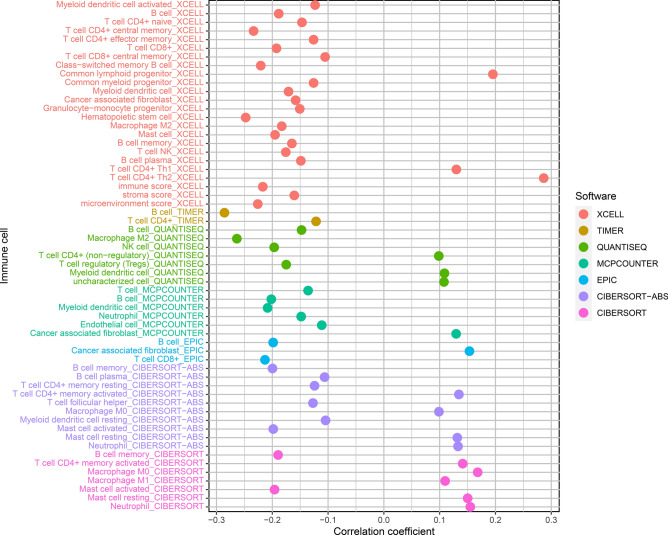
Positive and negative associations between immune-cell infiltration and risk score. Correlation coefficient > 0: positive correlation; Correlation coefficient < 0: negative correlation.

We presented data representing the link of the tumor immune microenvironment with the risk model (Fig. 7 and Supplementary Table 6). Spearman correlation analysis revealed that the high-risk group was strongly linked to the tumor-infiltrating immune cells, including T cell CD4 + Th1, Macrophage M0, Cancer-associated fibroblast, T cell CD4 + Th2, common lymphoid progenitors, uncharacterized cells, T cell CD4 + memory activated and neutrophils (Supplementary Fig. 1).

Aging is involved in the immune regulation of LUAD, including regulating T cell apoptosis, the T cell receptor signal pathway, cytotoxicity mediated by natural killer cells, and B cell-mediated immunity. T cells, macrophages, B cells, NK cells, and others participate in cancer immune surveillance through immune recognition and killing malignant cells. Immune cell infiltration in the tumor microenvironment contributes to tumor growth. The high-risk group has a higher immunoinvasive status and fewer cytotoxic lymphocyte infiltrates.

### Correlations between risk models and ICI-related genes

**Figure 8:**
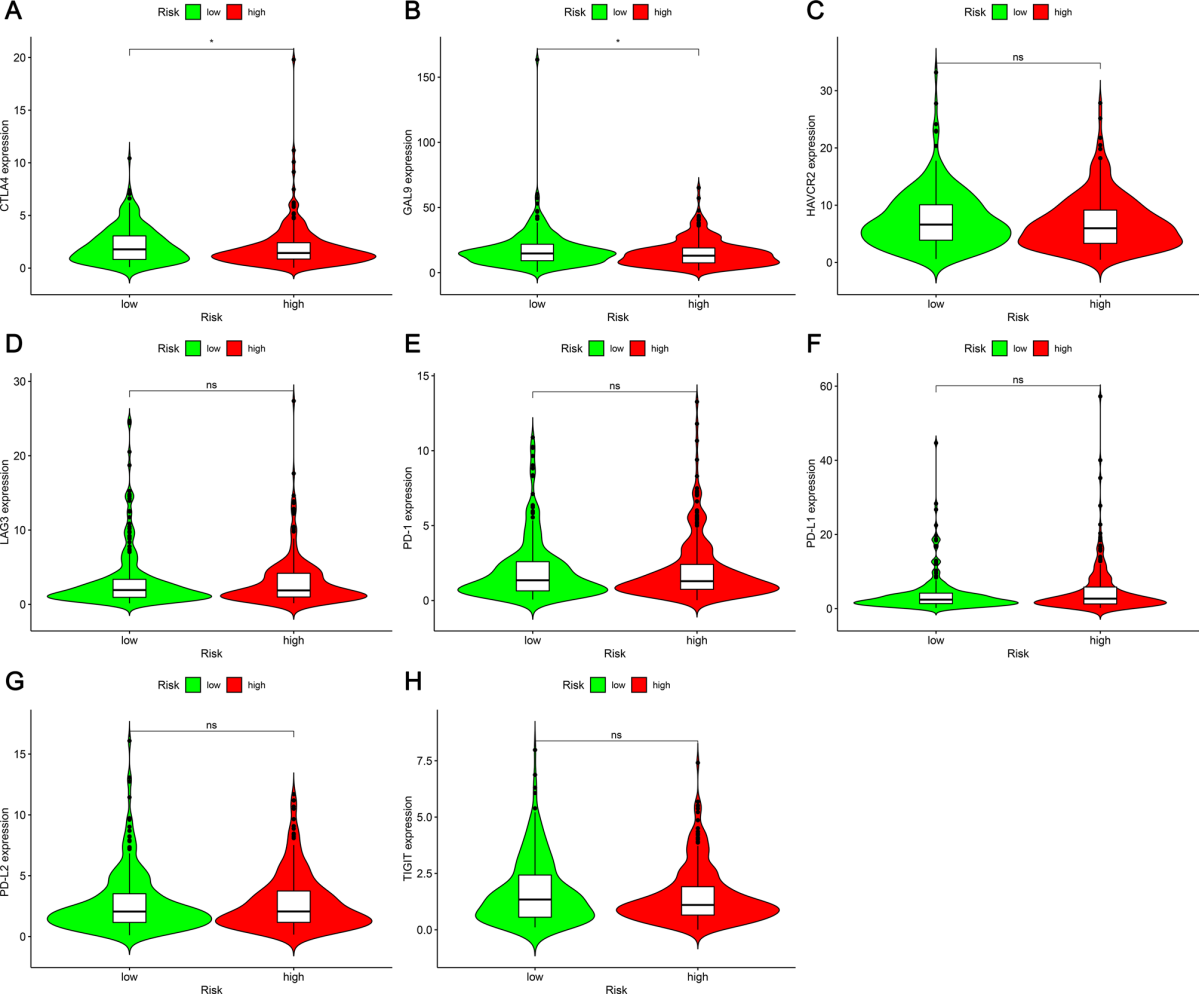
The link between ICI-related genes and risk model in LUAD. The levels of expression of (**A**) CTLA4; (**B**) GAL9; (**C**) HAVCR2; (**D**) LAG3; (**E**) PD1; (**F**) PD-L1; (**G**) PD-L2; (**H**) TIGIT in high-risk and low-risk groups. *Ns* not significant; *p < 0.05.

We investigated the correlation between the ICI-related gene and the risk model and found that the expression levels of CTLA4 and GAL9 differed between the groups (p < 0.05, Fig. 8A,B). The CTLA4 gene was subjected to upregulation in the high-risk group, while the level of the GAL9 gene was elevated in the low-risk group. These two genes can be used as immunotherapy targets in the high- and low-risk groups, respectively. HAVCR2 (p > 0.05, Fig. 8C), LAG3 (p > 0.05, Fig. 8D), PD-1 (p > 0.05, Fig. 8E), PD-L1 (p > 0.05, Fig. 8F), PD-L2 (p > 0.05, Fig. 8G) and TIGIT (p > 0.05, Fig. 8H) did not show significant differences.

### Correlation between risk model and chemotherapeutic drugs

**Figure 9:**
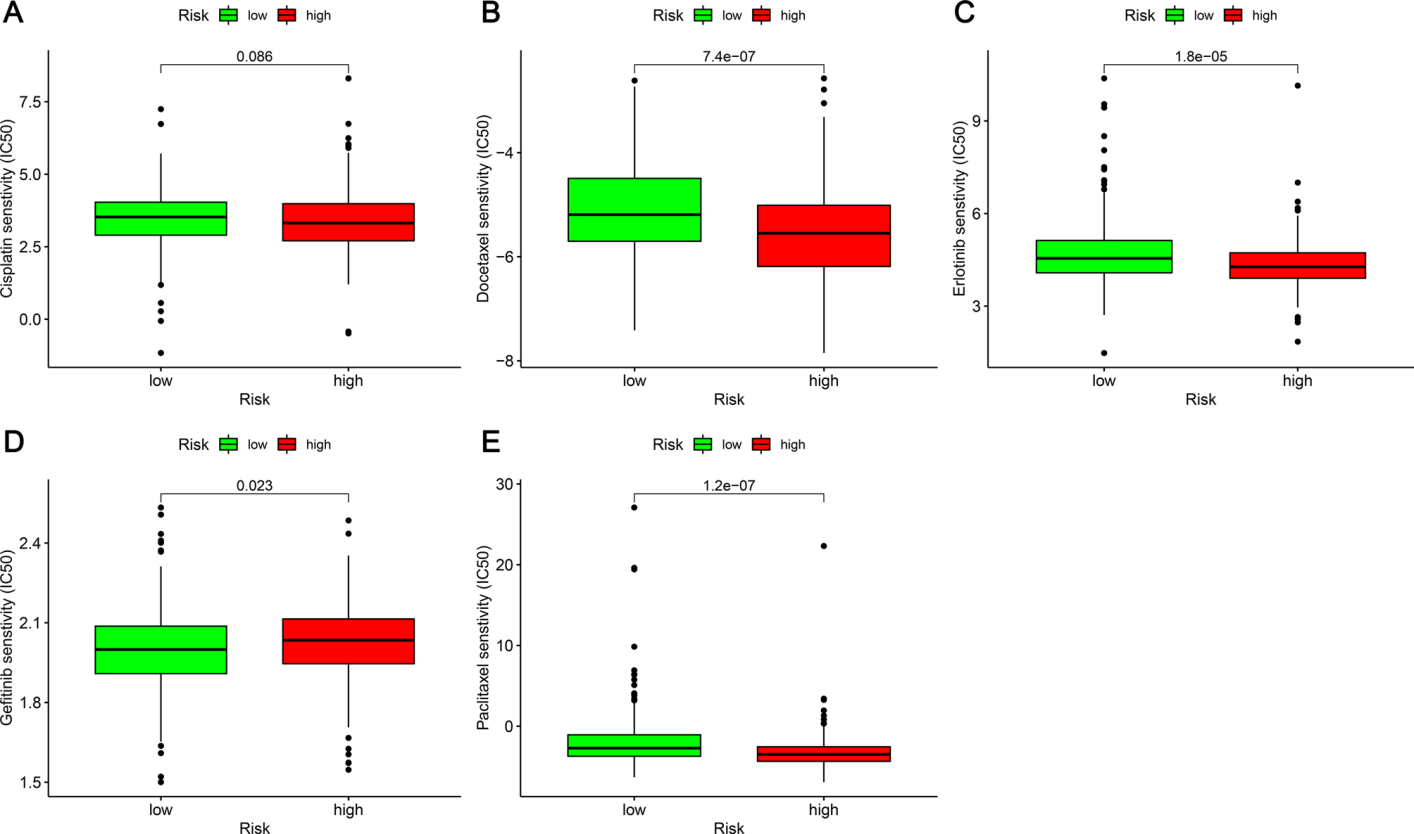
Correlation between risk model and chemotherapeutic drugs in LUAD. (**A**) cisplatin; (**B**) docetaxel; (**C**) erlotinib; (**D**) gefitinib, and (**E**) paclitaxel.

Docetaxel (Fig. 9B), erlotinib (Fig. 9C), gefitinib (Fig. 9D), and paclitaxel (Fig. 9E) showed significantly different sensitivities in the high- and low-risk groups, while cisplatin (Fig. 9A) showed no significant difference. In the high-risk group, the IC_50_ values of docetaxel, erlotinib, and paclitaxel were lower, indicating that these patients were sensitive to these drugs. Similarly, individuals in the low-risk group were sensitive to gefitinib.

The main mechanism of action of cisplatin is to interfere with the replication of tumor cell DNA, thereby killing tumor cells. It is a cyclical non-specific drug. Erlotinib is a tyrosine kinase inhibitor that reversibly inhibits specific types of epidermal growth factor receptor (EGFR) mutations. Docetaxel and paclitaxel are phase M cycle-specific agents. They inhibit cell mitosis by stabilizing and enhancing the polymerization of tubulin, preventing microtubule depolymerization. Gefitinib is a first-generation targeted drug for the treatment of lung adenocarcinoma, which blocks conduction by inhibiting EGFR's autophosphorylation and inhibits the proliferation of tumor cells. The different mechanisms of action of these drugs may be responsible for their differences in sensitivity in the high- and low-risk groups.

## Discussion

Aging can lead to metabolic disorders, malnutrition, and decreased immune function, triggering many chronic diseases. With age, the number of senescent cell divisions increases, and the probability of errors under the influence of external factors also increases significantly. The deterioration of the human immune system during aging and the reduced ability to monitor and kill cancer cells contributes to cancer development. Aging can also promote tumor development and metastasis^20,21^, risk factors for colorectal tumors and lung cancer^22^. A study found that ten serum proteins related to cervical cancer and tumor-specific proteins were closely related to aging^23^. Patients with higher expression of senescent cell-associated proteins had lower survival rates. Cancer symptoms improved by killing senescent cells and reducing the inflammatory environment, and survival rates increased. However, the association between LUAD and arlncRNA is poorly understood. We determined the probable function of arlncRNA to provide a basis for risk stratification and prognosis prediction in patients with LUAD.

There are several models related to aging. The evolution-based causation model takes the total and compositional causal model as a starting point and develops it using evolutionary and statistical theories^24^. It is used to develop an aging-related index and can fully explain the importance of genetics and environment to disease morbidity and mortality. This model has potential limitations. It applies to diseases with many sufficient causes and only to aging-related diseases and inherent mortality. The architecture proposed by Ye et al., called “Mashup and Deep Learning,” integrates multiple data sources^25^. It uses Mashup's algorithm to reduce the dimensionality of biological networks and proposes a modular deep neural network model that predicts genes associated with aging diseases. It provides an efficient multi-label learning algorithm to reduce the workload of experimental verification. One limitation is that few recommended genes result in a lack of evidence related to diseases. Xiao et al. designed two models based on a risk-scoring model and a clustering model of aging-related genes and glioma cases using LASSO-Cox regression analysis, consistent clustering analysis, and univariate Cox regression analysis^26^. It explored the expression profile, prognostic value, and potential role of aging-related genes in glioma. The study also explored the relationship between genetic mutations and risk scores, showing that the expression of aging-related genes can lead to gene mutations causing malignant diseases.

In the present study, we obtained LUAD patients’ ar-gene and lncRNA data from Human Aging Genomic Resources 3 and TCGA, analyzed differential co-expression establishing the DEarlncRNAs, and then performed lncRNA pair validation by cyclically single pairing them with a matrix of 0-or-1. Next, we acquired each sample’s risk coefficient of patients with LUAD and constructed a risk coefficient model by performing univariate regression, LASSO regression analyses, and Cox multivariate and univariate regression analyses. Third, we computed each ROC’s AUC to obtain the optimal model fit and obtained the cutoff value based on the Akaike information criterion. Then, we used it to identify patients in the high- and low-risk groups. We performed correlation analyses to assess the efficacy and accuracy of the risk model. We determined whether the two groups had substantial differences in the survival rate, correlation of clinical characteristics, tumor-infiltrating immune-cell expression, gene expression, and chemotherapeutic drug sensitivity. The findings suggested that the model works well and has strong predictive power.

Correlation analysis of risk scores with tumor-infiltrating immune cells suggested that the tumor microenvironment may be involved in tumorigenesis, including tumor-promoting or tumor-antagonizing. The immune evasion ability of cancer cells is a hallmark of tumor-related therapeutic strategies^27^. Xu et al. found that HK3 enhanced macrophages and monocyte infiltration that presented the antigens of the cell surface and modulated the debilitating T cells' critical genes (PD1 and CTLA4), affecting the process of immune escape^28^. We can use immune cells as therapeutic agents in fighting tumor cells. Immune checkpoint inhibitors such as anti-CTLA4 and anti-PD antibodies and adaptive immune cells such as CAR-T exert significant anticancer effects in several cancers^27^. We observed elevated expression levels of CTLA4 in samples from patients in the high-risk group, which serve as an effective therapeutic target for patients with LUAD.

Although we validated the new model in many ways, this study has several limitations. This was a retrospective study, and it may be biased as a result. The raw data were insufficient, the risk factor models lacked external validation on clinical datasets, and in-depth mechanistic studies were not performed. Therefore, we will integrate prospective, multi-center, real-world experimental data in the next step.

In conclusion, the risk model constructed by arlncRNAs can be used for risk stratification among patients with LUAD and as a novel marker to predict prognosis, helping to identify patients who may benefit from targeted and chemotherapeutic agents. The authors believe that the risk stratification and prognosis prediction model constructed by arlncRNAs can be extended to other tumors.

## Supplementary Information

Supplementary Legends.

Supplementary Figure 1.

Supplementary Table 1.

Supplementary Table 2.

Supplementary Table 3.

Supplementary Table 4.

Supplementary Table 5.

Supplementary Table 6.

## Data Availability

The datasets generated or analyzed during the current study are available at The Cancer Genome Atlas repository, [https://portal.gdc.cancer.gov/repository], [https://portal.gdc.cancer.gov/repository?facetTab=cases&filters=%7B%22op%22%3A%22and%22%2C%22content%22%3A%5B%7B%22op%22%3A%22in%22%2C%22content%22%3A%7B%22field%22%3A%22cases.primary_site%22%2C%22value%22%3A%5B%22bronchus%20and%20lung%22%5D%7D%7D%2C%7B%22op%22%3A%22in%22%2C%22content%22%3A%7B%22field%22%3A%22cases.project.program.name%22%2C%22value%22%3A%5B%22TCGA%22%5D%7D%7D%2C%7B%22op%22%3A%22in%22%2C%22content%22%3A%7B%22field%22%3A%22cases.project.project_id%22%2C%22value%22%3A%5B%22TCGA-LUAD%22%5D%7D%7D%2C%7B%22op%22%3A%22in%22%2C%22content%22%3A%7B%22field%22%3A%22files.analysis.workflow_type%22%2C%22value%22%3A%5B%22STAR%20-%20Counts%22%5D%7D%7D%2C%7B%22op%22%3A%22in%22%2C%22content%22%3A%7B%22field%22%3A%22files.data_category%22%2C%22value%22%3A%5B%22transcriptome%20profiling%22%5D%7D%7D%2C%7B%22op%22%3A%22in%22%2C%22content%22%3A%7B%22field%22%3A%22files.data_type%22%2C%22value%22%3A%5B%22Gene%20Expression%20Quantification%22%5D%7D%7D%5D%7D].

## Abbreviations

LUAD: Lung adenocarcinoma
lncRNA: Long non-coding RNA
arlncRNAs: Aging-related long non-coding RNAs
DEarlncRNAs: Differently expressed aging-related long non-coding RNAs
AUC: Area under the curve
ICIs: Immune checkpoint inhibitors
ar-genes: Aging-related genes
IC_50_: Half-inhibition rate
ICB: Immune checkpoint blockade
RNAseq: Transcriptome Akaike information criterion profiling
ROC: Receiver operating characteristics
LASSO: Least absolute shrinkage and selection operator
uni-Cox: Univariate Cox
CI: Confidence interval
GTF: Gene transfer format
TCGA: The Cancer Genome Atlas
HR: Hazard ratio
EGFR: Epidermal growth factor receptor
