# Supplementary material for: A risk stratification and prognostic prediction model for lung adenocarcinoma based on aging-related lncRNA
Source: Sci Rep. 2023 Jan 10;13:460. doi: 10.1038/s41598-022-26897-2 (PMC9832126; doi:10.1038/s41598-022-26897-2)
Supplement: Supplementary file 1 — Supplementary Legends. [file 41598_2022_26897_MOESM1_ESM.docx]

**Additional files**

Supplementary Table 1. Aging-related genes list.

Supplementary Table 2. Aging-related lncRNAs list.

Supplementary Table 3. Dysregulated aging-related lncRNAs in LUAD.

Supplementary Table 4. 4365 differentially expressed aging-related lncRNAs.

Supplementary Table 5. Aging-related lncRNAs that significantly affected the prognosis in LUAD.

Supplementary Table 6. Immune-cell infiltration based on XCELL, QUANTISEQ, TIMER, CIBERSORT, EPIC, MCPCOUNTER, and CIBERSORT-ABS.

Supplementary Figure 1. Immune-cell infiltration in high- and low-risk LUAD samples.
