# Supplementary figures and images for: A risk stratification and prognostic prediction model for lung adenocarcinoma based on aging-related lncRNA
Source: Sci Rep. 2023 Jan 10;13:460. doi: 10.1038/s41598-022-26897-2 (PMC9832126; doi:10.1038/s41598-022-26897-2)

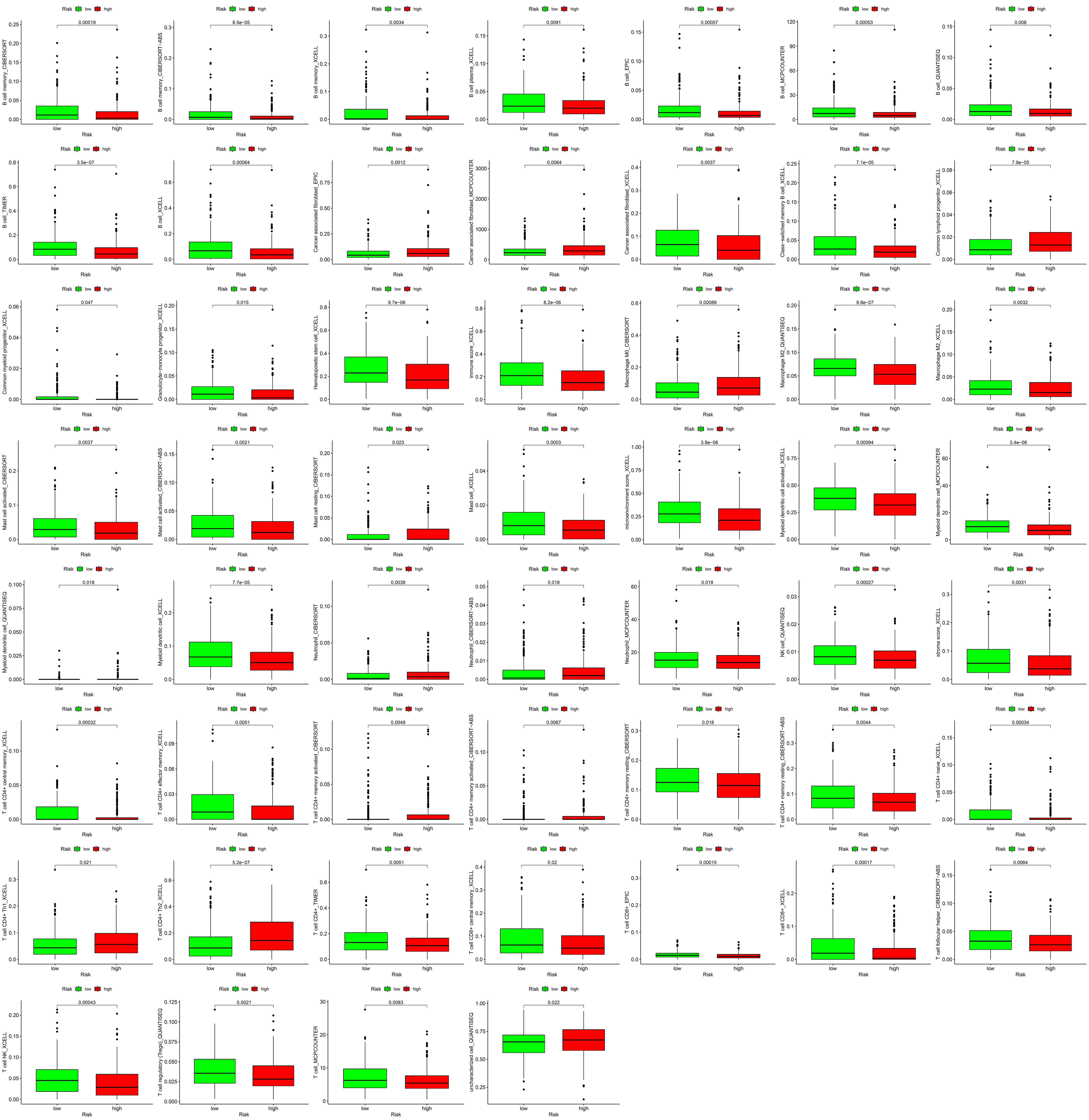

Supplement: Supplementary file 2 — Supplementary Figure 1. [file 41598_2022_26897_MOESM2_ESM.tif]
